# Doppler-inclusive reclassification of fetal growth restriction based on Japanese criterion: a single-center study on perinatal outcomes of pathological and normal small fetuses

**DOI:** 10.1007/s10396-025-01565-w

**Published:** 2025-10-04

**Authors:** Sumito Nagasaki, Hikari Kotaki, Makiko Shimabukuro, Junya Sakuma, Mayumi Takano, Masahiko Nakata

**Affiliations:** 1 Department of Obstetrics and Gynecology, Toho University Omori Medical Center, 6-11-1 Omorinishi, Ota-ku, Tokyo, 143-8541 Japan; 2 Department of Obstetrics and Gynecology, Toho University Faculty of Medicine, 5-21-16 Omorinishi, Ota-ku, Tokyo, 143-8541 Japan

**Keywords:** Fetus, Fetal ultrasound, Doppler, Fetal growth restriction

## Abstract

**Purpose:**

Criteria for diagnosing fetal growth restriction (FGR) vary globally. The Japanese criterion is estimated fetal weight (EFW) below − 1.5 standard deviations, without distinctions based on gestational age or severity. However, some international diagnostic criteria classify FGR using gestational age, Doppler assessments, and growth. While the Japanese criterion is simple and easy to apply, including EFW, gestational age at diagnosis, growth, and Doppler findings can provide a more comprehensive assessment of fetoplacental function. The aims of this study were: [1] to reclassify small fetuses on the Japanese criterion into early FGR, late FGR, or small for gestational age (SGA) groups by applying Doppler-inclusive diagnostic criteria, and [2] compare the perinatal outcomes.

**Methods:**

In this retrospective study, FGR diagnosed based on the Japanese criterion between 2017 and 2021 at our hospital were reclassified into early FGR, late FGR, or SGA by applying Doppler-inclusive criteria. Cases not classified as early or late FGR were categorized as SGA. Perinatal, maternal, and neonatal outcomes were analyzed across all groups.

**Results:**

Overall, 184 growth-restricted fetuses based on the Japanese criterion—160 cases (42 early FGR, 51 late FGR, and 67 SGA)—were enrolled after excluding 24 cases of fetal malformation. Gestational age at delivery, mode of delivery, and maternal and neonatal complications differed significantly among the groups. The early FGR group showed a significantly higher incidence of preterm birth, emergent Cesarean section, and severe maternal or neonatal complications.

**Conclusions:**

FGR with Doppler-inclusive criteria can be better for practical use providing high relevance to perinatal outcome.

## Introduction

Fetal growth restriction (FGR) is defined as the failure of the fetus to meet its growth potential due to pathological factors, most commonly placental dysfunction [1]. Compared with normally developing fetuses, FGR is a major contributor to stillbirth, neonatal mortality, and short- and long- term morbidity [2, 3]. On the other hand, some healthy fetuses are smaller than expected for their gestational age, despite being within the normal growth potential. This condition is known as small for gestational age (SGA). The diagnostic criteria for SGA and FGR and timing for evaluation shows variation among countries and academic societies [1, 2, 4–15]. In Japan, FGR is diagnosed using standard estimated fetal weight (EFW) values [16], with a cutoff of −1.5 standard deviations (SD). Serial evaluation of EFW, fetal abdominal circumference, and amniotic fluid volume are usually considered for estimating the pathophysiology [17] (Table 1A). This Japanese criterion offers a simple and effective screening method but fails to distinguish the severity or onset period of growth restriction. In contrast, some international guidelines [1, 10] distinguish FGR from SGA by assessing both fetal size estimation and Doppler findings. Furthermore, they also classify FGR into early FGR (before 32 weeks) and late FGR (after 32 weeks) (Table 1B). These Doppler-inclusive criteria seem more complex than the Japanese one, but they bring advantage to assess the fetoplacental circulation. In this retrospective study, we attempted to reclassify FGR according to the Japanese criterion into early FGR, late FGR, and SGA groups by applying additional Doppler assessment, severity of smallness of EFW or AC, fetal growth, and gestational age at diagnosis, which allowed us to investigate the difference in perinatal morbidity and outcomes.

**Table 1:**
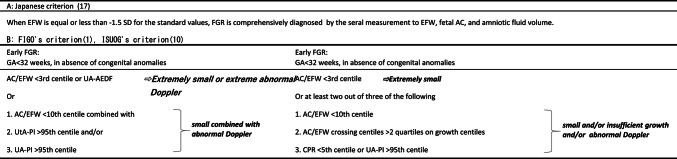
Japanese criterion and Dopler-Inclusive criteria

EFW, estimate fetal weight; AC, abdominal circumference; FGR, fetal growth restriction GA: gestational age; UA-AEDF, duration of absent end-diastolic umbilical artery flow; UtA-PI, uterine artery pulsatility index; UA-PI, umbilical artery pulsatility index; CPR, cerebroplacental ratio

## Methods

### Study design

A retrospective case-control single-center study was carried out. Japanese expecting mothers with FGR fetuses according to the Japanese criterion diagnosed at our hospital between 2017 and 2021 were investigated. The Japanese criterion and other ones including severity of smallness of EFW or AC, fetal growth, gestational age at diagnosis, and Doppler analysis are shown in Table 1. Criteria (B) were based on the International Society of Ultrasound in Obstetrics and Gynecology (ISUOG) [10] and International Federation of Gynecology and Obstetrics (FIGO) [1] guidelines. All cases were verified to determine whether they met the diagnostic criteria for FGR based on either of the criteria (B) throughout pregnancy. Specifically, medical records were checked following the progression of pregnancy from the earlier gestational age until delivery. Since there is a difference between early FGR and late FGR based on criteria (B), the first step was to determine whether they met the criteria for early FGR before 32 weeks in all cases. When a case did not meet the criteria for early FGR, additional assessment was made if the case met the criteria for late FGR after 32 weeks. In this study, when a case did not meet the criteria for either early FGR or late FGR, we classified it as SGA. Fetuses with chromosomal or structural abnormalities were excluded in accordance with the ISUOG definitions. This study intended to focus on fetuses diagnosed with FGR using the Japanese criterion. We could not enroll fetal cases with estimated weight between − 1.5 and − 1.28 SD (the upper threshold of ISUOG’s SGA criterion (10th percentile)), because of the methodological issue for recruiting cases in this study. Therefore, the SGA group reclassified in this study is slightly different from the original SGA group defined by ISUOG and FIGO.

We assessed perinatal, maternal, and neonatal outcomes in this study. Evaluated factors included obstetric history, assisted reproductive technology (ART), maternal age at delivery, maternal height and body weight, body mass index (BMI) before pregnancy, gestational age at delivery, preterm birth rate, mode of delivery, neonatal birth weight, neonatal height, neonatal head circumference, umbilical artery blood pH, Apgar score (Ap), and ultrasound parameters such as umbilical artery pulsatility index (UA-PI), middle cerebral artery pulsatility index (MCA-PI), cerebroplacental ratio pulsatility index (CPR-PI), and uterine artery pulsatility index (UtA-PI). Positive maternal complications were defined if any of the following were observed: severe (≧ 160/110 mmHg)hypertensive disorders of pregnancy (HDP), preeclampsia, HELLP syndrome, placental abruption, and eclampsia. Severe neonatal complications included intraventricular hemorrhage (grade I or higher), periventricular leukomalacia (grade I or higher), sepsis, necrotizing enterocolitis, meconium-related ileus requiring surgery, retinopathy of prematurity (ROP) (grade II or higher), chronic lung disease, and death. Mild neonatal complications included hypoglycemia, polycythemia, anemia of prematurity, leukopenia, thrombocytopenia, and respiratory distress syndrome.

### Perinatal management

We performed serial ultrasound examinations at least once a week to evaluate the fetal growth and Doppler findings. Based on the gestational age and the fetal condition, additional close ultrasound, fetal heart rate monitoring, and biophysical profile scoring (BPS) were performed. A single course of antenatal corticosteroids was administrated to accelerate fetal lung maturation in cases where there was a risk for preterm birth before 34 weeks within the next 2 weeks. The decision of delivery at our hospital was made by referring to the Japanese clinical guidelines [17] and the international management methods indicated by ISUOG [3]; non-reassuring fetal status such as decreased BPS, or repeated decelerations observed on cardiotocography, critical abnormal Doppler in umbilical artery and/or ductus venosus and cerebroplacental ratio. Artificial birth was actively intended when maternal severe complications such as severe HDP, HELLP syndrome, placental abruption, or preeclampsia had been observed. When extension of the gestation period was deemed possible, we awaited spontaneous labor. Elective cesarean sections were performed at 38 weeks. We did not evaluate short-term variability because it is not widely available in Japan. When a vaginal delivery could be expected, we did so, but in cases where an emergent delivery was necessary due to fetal or maternal condition, we opted for cesarean section. For all preterm infants born before 34 weeks, we perform ultrasound and MRI examinations. For other infants, these examinations are conducted by neonatologists as needed. All neonatal diagnoses were made by neonatologists. Additionally, we analyzed whether perinatal outcomes differed among subgroups based on Doppler ultrasound assessment even before 32 weeks. The subgroups consisted of early SGA (diagnosed before 32 weeks) and late SGA (diagnosed after 32 weeks).

### Statistical analysis

We compared outcomes across three groups in the statistical analyses. The Shapiro-Wilk test was employed to assess the normality of data distribution. For non-normally distributed data, the Kruskal-Wallis test, χ^2 ^test, and Mann-Whitney U test were applied, with a Steel-Dwass test used for post-hoc pairwise comparisons among groups where significant differences were identified. The statistical significance level was set at *p* = 0.05. Data were analyzed using Bell Curve for Excel (Social Survey Research Information Co., Ltd.).

## Results

**Fig. 1:**
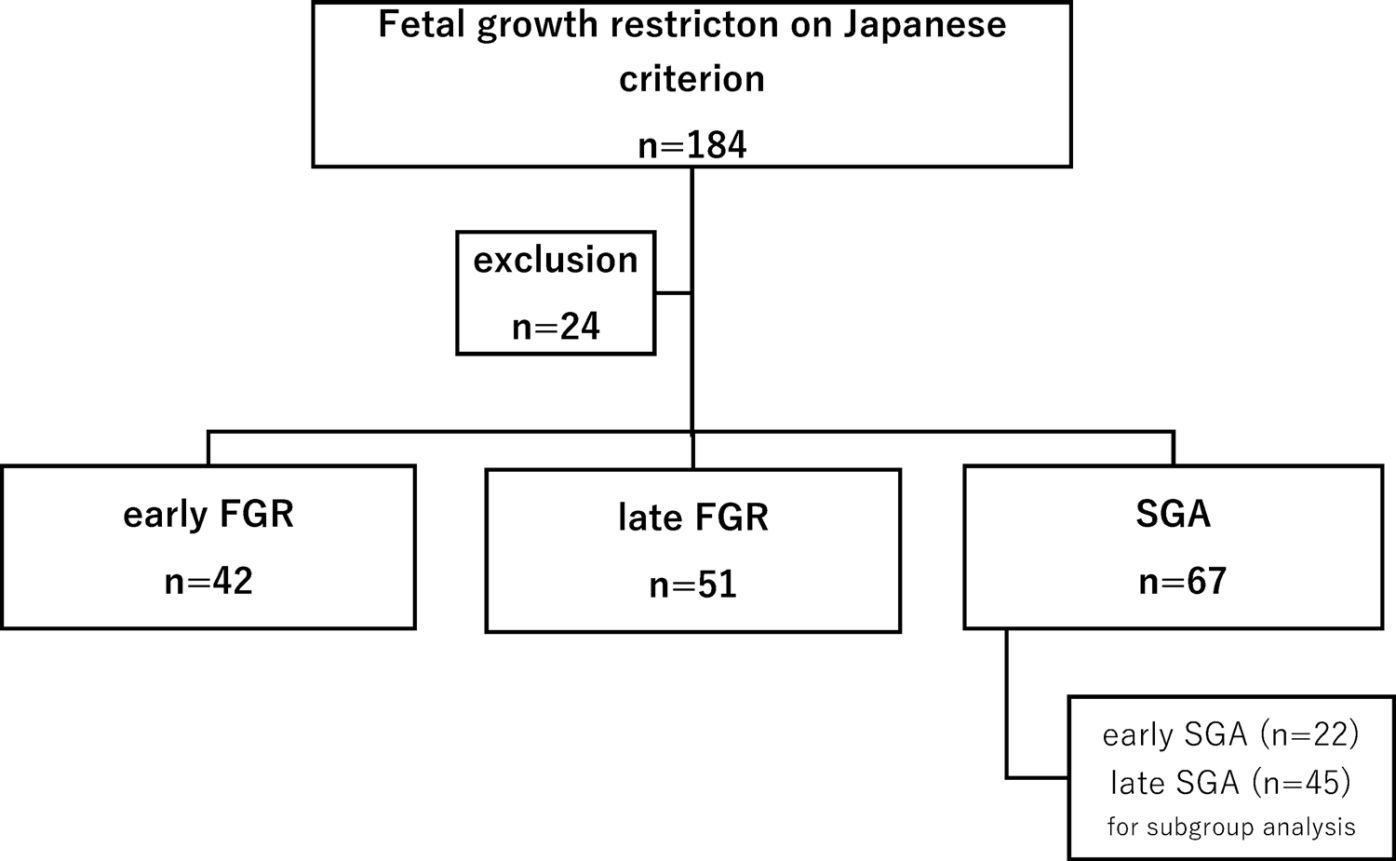
Reclassification flowchart of fetal growth restriction according to the Japanese criterion into three groups and one subgroup based on Doppler-inclusive criteria. FGR, fetal growth restriction; SGA, small for gestational age

**Fig. 2:**
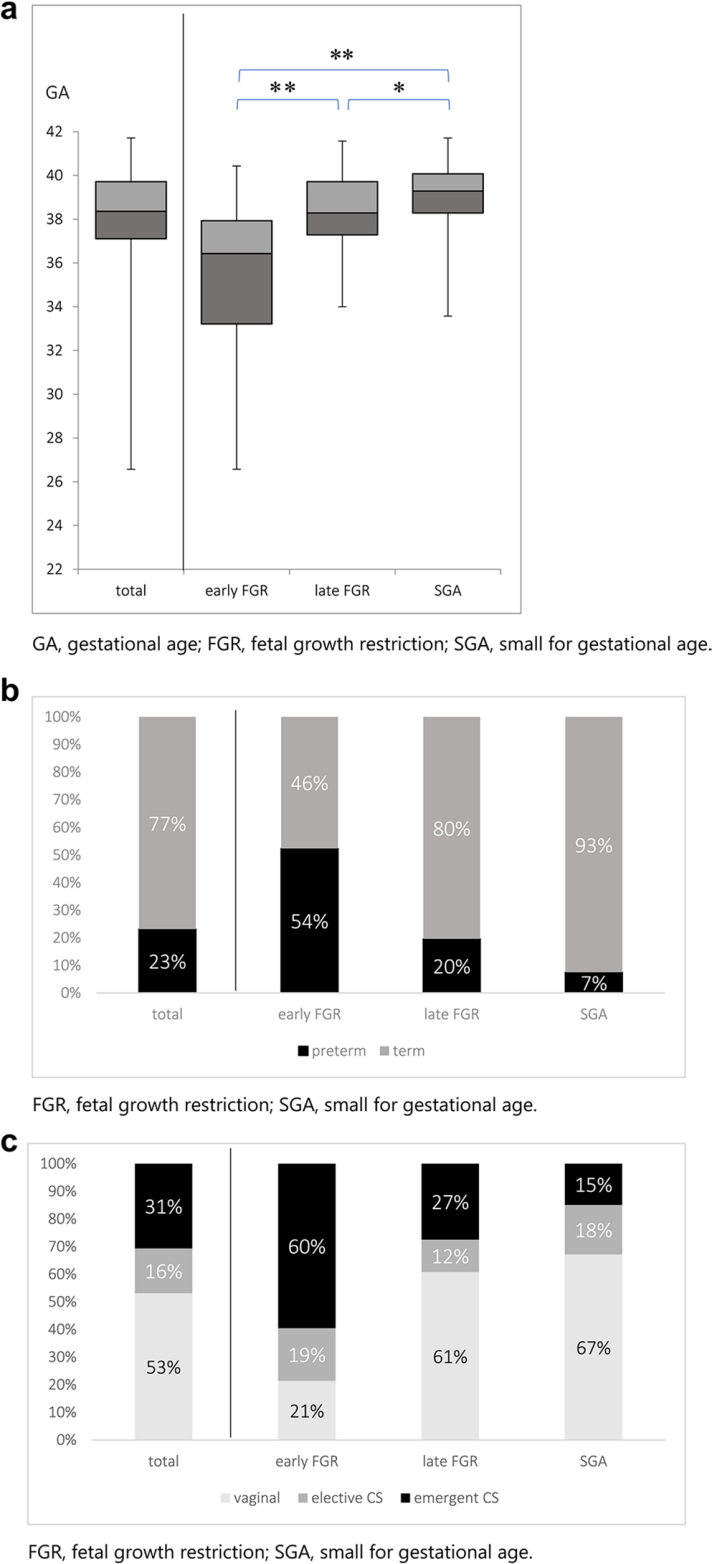
(**a**) Box-and-whiskers plots of gestational age at delivery in early FGR, late FGR, and SGA, (**b**) Rate of preterm birth in early FGR, late FGR, and SGA, (**c**) Mode of delivery in early FGR, late FGR, and SGA. FGR, fetal growth restriction; SGA, small for gestational age

A total of 184 patients were diagnosed with FGR according to the Japanese criterion during the study period. Ultimately, 160 cases were enrolled after excluding 24 cases of fetal malformation. Reclassification resulted in 42 cases in the early FGR group, 51 in the late FGR group, and 67 in the SGA group (Fig. 1). The median gestational age at delivery of these 160 cases, along with the first and third quartiles, was 38.4 weeks (37.1–39.7) (Fig. 2a). The preterm birth rate, vaginal delivery rate, elective cesarean section rate, and emergent cesarean section rate were 23%, 53%, 16%, and 31%, respectively (Fig. 2b and c). The median gestational age at diagnosis was 28 (25–30) in the early FGR group, 35 (34-36.7) in the late FGR group, and 34 (30.25-36) in the SGA group. As the data for each group were not normally distributed, the Kruskal-Wallis, Steel-Dwass, Mann-Whitney U, and χ^2^ tests were used for analysis.

Maternal characteristics are summarized in Table 2. Parity, ART, maternal age at delivery, and maternal height were not significantly different among the three groups. Pre-pregnancy body weight and pre-pregnancy BMI were significantly lower in the late FGR group compared to the early FGR group but not significantly different from the SGA group. The ultrasound findings at diagnosis are summarized in Table 3. The UA-PI and CPR-PI were significantly different, which is consistent with the diagnostic criteria. Figure 2a, b, and c show the delivered weeks, the rate of preterm birth, and the delivery mode of the three groups, respectively. Gestational age at delivery was significantly different among the three groups, with the median interquartile range (IQR) for the early FGR group being 36.4 (33.2–37.9) weeks (Fig. 2a). The preterm birth rate was significantly higher in the early FGR group (> 50%) (Fig. 2b). The early FGR group had the lowest rate of vaginal delivery and the highest rate of emergent cesarean section among the groups (Fig. 2c). The neonatal characteristics are summarized in Table 4. Birth weight percentile and head circumference percentile in the SGA group were significantly higher than those in both the early FGR and late FGR groups. The pH of umbilical artery blood and Ap at delivery showed no differences, and Ap was significantly lower in the early FGR group. Maternal and neonatal complications are summarized in Table 5. The incidence of severe maternal and severe neonatal complications was significantly higher in the early FGR group than in the other groups. We had only two cases of severe neonatal complications in the early FGR group, which were delivered at 26 and 29 weeks. In the subgroup analysis distinguishing between early SGA and late SGA, a significant difference was observed in both early SGA and early FGR (Table 6).

**Table 2:**
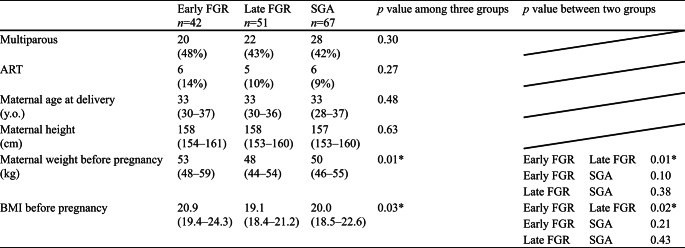
Maternal characteristics of study population

Data are shown as n (%) or median (IQR). Kruskal-Wallis test or χ2 test for evaluation in three groups and Steel-Dwass for testing in two groups. ART, Assisted Reproductive Technology; BMI, body mass index

**Table 3:**
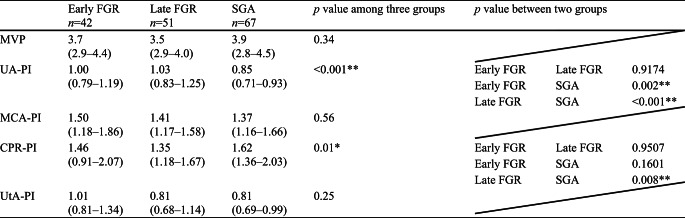
Ultrasound findings at diagnosis

Data are shown median (IQR). Kruskal-Wallis used for testing in three groups and Steel-Dwass for testing in two groups. MVP, maximum vertical pocket; UA-PI, umbilical artery pulsatility index; MCA-PI, middle cerebral artery pulsatility index; CPR-PI, cerebroplacental ratio pulsatility index; UtA-PI, uterine artery pulsatility index

**Table 4:**
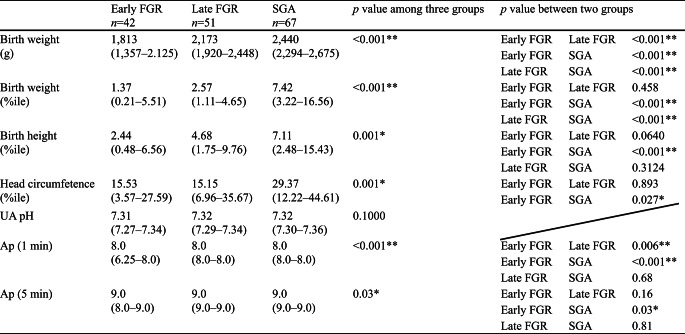
Comparison of neonatal data in three groups

Data are shown n(%) or median(IQR). Kruskal-Wallis or χ2 test for testing in three groups and Steel-Dwass or χ2 test for testing in two groups. ART, Assisted Reproductive Technology; BMI, body mass index. UA, umbilical artery

**Table 5:**
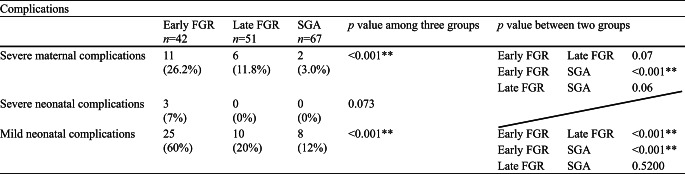
Maternal and neonatal complications

Data are shown number (%) or median (IQR). Kruskal-Wallis or χ2 test for testing in three groups and Steel-Dwass or χ2 test for testing in two groups

**Table 6:** Subgroup analysis of early SGA, early FGR and late SGA

	*n*:22	*n*:43		*n*:45
	Early SGA	Early FGR	*p* value	Late SGA
GA at diagnosis	27.4	28.0	n.s.	35.1
Birth weight	2,530	1,813	<0.001**	2430
GA at delivery	38.9	36.4	<0.001**	39
Preterm birth	14%	54%	<0.001**	0.40%
Vaginal delivery	67%	21%	<0.001**	69%
Emargent CS	14%	60%	<0.001**	16%
Severe maternal complication	0.45%	26%	<0.001**	0.90%
Severe newborn complication	0%	7%	n.s.	0%

Data are shown n(%) or median(IQR). Kruskal-Wallis or χ2 test for testing in three groups and Steel-Dwass or χ2 test for testing in two groups. ART, Assisted Reproductive Technology; BMI, body mass index. UA, umbilical artery

## Discussion

The first main finding was that the gestational age at delivery and the mode of delivery were significantly different (Fig. 2a, b, c). About a quarter of the early FGR group were delivered before 34 weeks. Conversely, about three-quarters of the late FGR and SGA groups were delivered after 37 weeks (Fig. 2a). The major delivery mode in the early FGR group was emergent cesarean section, in 60% of the patients (Fig. 2c), mainly due to non-reassuring fetal status and severe HDP. In contrast, over 60% of the late FGR and SGA cases were delivered vaginally. Although this was a retrospective study, the results demonstrated that even in cases diagnosed using the Japanese criterion of an estimated fetal weight below − 1.5 SD, perinatal outcomes differed when classified into SGA, early FGR, and late FGR. It can be crucial to recognize that SGA fetuses are at less risk for adverse perinatal outcomes than FGR cases. An association between MCA vasodilatation or the alteration of its ratio with UA-PI and poorer perinatal outcomes has previously been reported [18]. Baschat also reported that in FGR, an increase in Doppler indices and brain-sparing occur prior to the deterioration of the BPS [19]. Furthermore, in addition to Doppler findings, the evaluation of growth arrest, defined as AC or EFW crossing more than two quartiles on growth centiles, is particularly important in the assessment of late FGR [1, 9]. The perinatal outcomes of SGA, early FGR, and late FGR in this study highly suggest an advantage to classify small fetuses not only by EFW but also using additional Doppler analysis, gestational age, and fetal growth. For instance, early FGR cases in this study had the highest risk of requiring emergent cesarean sections in the preterm period, while SGA cases appear to have a relatively lower risk of adverse perinatal outcomes compared to the other groups. The subgroup analysis comparing early SGA and early FGR also demonstrated significant differences in the perinatal outcomes, which suggested that Doppler-inclusive assessment is essential without relation to the gestational age.

The second main finding was that maternal and neonatal complications were significantly different among the groups. The early FGR group showed significantly higher severe maternal complications, including severe HDP, HELLP syndrome, placental abruption, and eclampsia (26.2%) compared to 11.8% in the late FGR group and 3% in the SGA group (Table 5). Severe maternal complications in early FGR were reported to have associations with HDP and placental insufficiency [20]. Regarding severe neonatal complications, there were only two cases in the early FGR group, which were delivered at 26 and 29 weeks. The higher incidence of maternal and neonatal complications in the early FGR group was clinically significant.

Additionally, birth weights were significantly different among the three groups. However, birth weight is related to the gestational age at delivery. The birth weight percentile, as a standardized value accounting for gestational age, showed no difference between early FGR and late FGR but was significantly higher in the SGA group. Apgar scores were significantly lower in the early FGR group. This lower Apgar score in the early FGR group may be responsible for prematurity with the earlier gestational age at delivery, even though there were no differences in umbilical arterial blood pH among the groups.

There were several limitations in this study. First, it was a single-center retrospective study; therefore, the sample size was limited. Additionally, we could not enroll fetuses with estimated weight between the 10th percentile (≒−1.28 SD) and − 1.5SD since we recruited cases with EFW equal or less than − 1.5 SD, according to the Japanese criterion. Therefore, the characteristics of the SGA group in this study may differ slightly from those originally defined by ISUOG and FIGO. One of the unique approaches in this study was to divide SGA into subgroups based on gestational age.

In conclusion, while the current Japanese criterion is highly effective for screening and plays an important role in identifying high-risk cases, additional Doppler examination is thought to offer considerable benefits in perinatal management, clinical practice, and research.

## Data Availability

The data that support the findings of this study are available on request from the corresponding author. These data are not publicly available due to privacy or ethical restrictions as they include information such as maternal height, maternal weight, birth weight, and gestational age at delivery.
